# Trigeminal Rhizotomy Using Gyroscopic Radiosurgery: A Case Report

**DOI:** 10.7759/cureus.24951

**Published:** 2022-05-12

**Authors:** Peter D Klassen, Sai K Ivaturi, Benjamin K Hendricks

**Affiliations:** 1 Neurosurgery, Bonifatius Hospital, Lingen, DEU; 2 Clinical Applications Engineering, Zap Surgical Systems, Inc., San Carlos, USA; 3 Neurosurgery, Barrow Neurological Institute, Phoenix, USA

**Keywords:** posterior fossa, novel, zap, neuralgia, gyroscopic, stereotactic radiosurgery

## Abstract

Characterized by intense, episodic lancinating pain within the distribution of the trigeminal nerve, trigeminal neuralgia (TN) is the most common craniofacial pain syndrome. Failure of medical management requires the consideration of interventional procedures. Stereotactic radiosurgery (SRS) is one of the more commonly used surgical options. Herein, we report the first published case of a patient with TN treated in the ZAP-X (San Carlos, CA: ZAP Surgical Systems, Inc.) gyroscopic radiosurgery system. This 59-year-old man with multiple sclerosis and recurrent intractable left idiopathic TN following previous SRS was retreated in the Zap-X system using 100 isocentric 5 mm beams to a dose of 7500 cGy. At a three-month follow-up, the patient reported a 45% decrease in his visual analogue scale (VAS) and a reduced need for medication. Albeit preliminary, this initial experience highlights the feasibility of a self-shielded, cobalt-free, device for radiosurgically treating TN.

## Introduction

Characterized by sudden episodes of paroxysmal stabbing, shock-like or burning pain within one or multiple divisions of the trigeminal nerve, trigeminal neuralgia (TN) is the most common craniofacial pain syndrome with a global incidence of four to five per 100,000 [1-3]. When refractory to medical management, TN generally warrants surgical intervention through either microsurgical vascular decompression, stereotactic radiosurgery (SRS), or trigeminal rhizotomy. Given its effectiveness and non-invasive nature, SRS is a particularly appealing alternative for patients with surgical comorbidities or advanced age. Moreover, radiosurgery tends to be associated with lower rates of re-treatment than other commonly used interventions, such as microvascular decompression, glycerol rhizotomy, radiofrequency ablation, and percutaneous balloon compression of the trigeminal ganglion [4]. These factors coupled with the increasing availability of SRS technology have driven the wider clinical adoption of radiosurgical rhizotomy over the past decade.

The goal of trigeminal rhizotomy is to downregulate (interrupt) nociceptive ephaptic nerve transmission via precision irradiation of the retrogasserian cisternal nerve segment. Ideally, this objective can be accomplished without significantly affecting facial sensation [1,2]. Among all conditions treated with SRS, the technical demands of treating TN are especially exacting; a very high radiation dose (60-120 Gy) must be delivered to a very small target immediately adjacent to highly critical anatomy, i.e., the brainstem. Maximal targeting accuracy and sharp dose falloff are mandatory [5]. While frame-based targeting was once felt to be essential, Romanelli et al. have demonstrated that the requisite precision can also be achieved with image-guided (non-frame-based) localization [1,2].

The ZAP-X (San Carlos, CA: ZAP Surgical Systems, Inc.) surgical robot is the first of its kind of gyroscopic radiosurgery (GRS) device dedicated to intracranial and head and neck applications. The Zap-X’s unique compact design enables significant solid angle coverage thereby ensuring a steep dose gradient. Meanwhile, by virtue of being “self-shielded,” a costly radiotherapy vault is not required [6]. Intra-treatment image guidance is provided by planar kV image acquisition and image-to-image matching with digitally reconstructed radiographs (DRRs). As an additional safety feature, Zap-X treatment is monitored with an integrated megavoltage (MV) detector that independently verifies dose delivery during treatment.

Initial clinical reports have demonstrated the efficacy of the Zap-X for treating intracranial and head and neck neoplasms [7,8]. However, some of its technical attributes also seem well-suited for treating TN. Of note, a short (45 mm) source to axis distance (SAD), solid angle coverage of 2π or greater, and small-aperture collimators (including both 4 mm and 5 mm diameters) collectively provide an especially steep dose gradient [5,6]. Moreover, the 1500 MU/min dose rate of the Zap-X LINAC enables relatively fast treatment. Either a single shot (Gamma Knife style; Stockholm, Sweden: Elekta AB) or inverse plan on a contoured target (Romanelli method) is possible with the Zap-X. In aggregate, these attributes make the Zap-X theoretically attractive for treating TN.

## Case presentation

History

**Figure 1:**
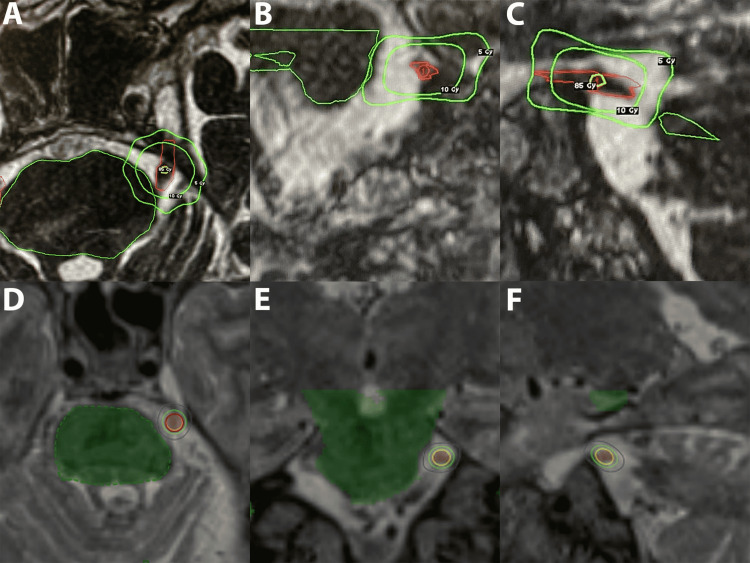
Treatment planning dose distribution graphics. Dose distribution for GKRS (A-C) and GRS (D-F) is illustrated on MRI within axial view (A and D), coronal view (B and E), and sagittal view (C and F). For GKRS, the outer green line is a 500 cGy isodose line, inner green is 1000 cGy isodose line, and yellow is the 8500 cGy isodose line (A-C). For GRS, the blue line is 2500 cGy isodose line, green is 5000 cGy isodose line, red is the target contour, and yellow is prescription 7500 cGy isodose line (D-F). MRI: magnetic resonance imaging; GKRS: Gamma Knife radiosurgery; GRS: gyroscopic radiosurgery

After an 11-year history of multiple sclerosis, a 59-year-old male presented with lancinating left-sided cheek and upper lip pain diagnosed to be TN involving the V2 division. The absence of vascular compression on magnetic resonance imaging (MRI) reinforced the diagnosis of idiopathic TN secondary to multiple sclerosis. Upon failing multimodal medical therapy, including narcotics and antiepileptic drugs, the patient was offered a radiosurgical rhizotomy. This was performed with Gamma Knife radiosurgery (GKRS) utilizing a single isocenter with a 4 mm collimator and 85 Gy prescribed to the 95% isodose line (Figure 1, panels A-C).

**Figure 2:**
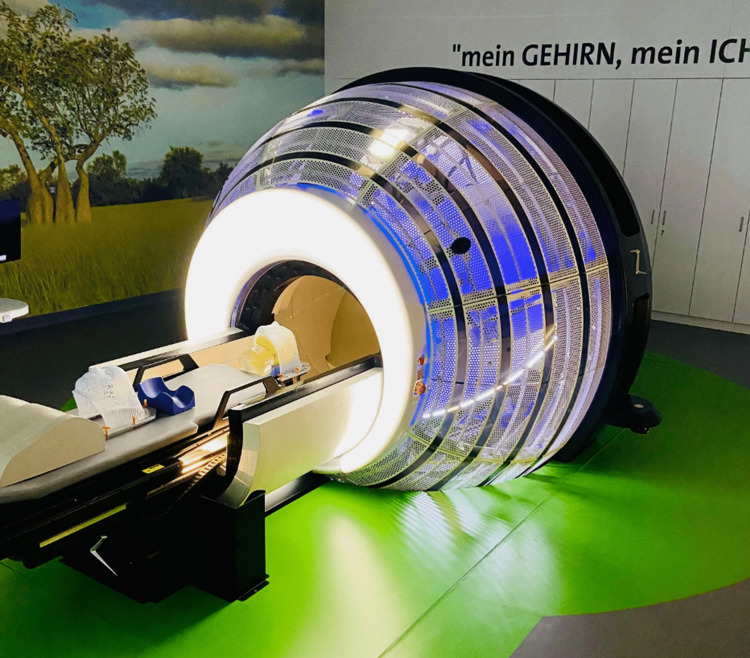
ZAP-X (San Carlos, CA: ZAP Surgical Systems, Inc.) gyroscopic radiosurgery system. This is the system present at Lingen, Germany, where the reported intervention was completed.

Two months post-GKRS, the patient experienced a decrease in his visual analog scale (VAS) from nine to seven while continuing medical therapy. Three months later, he suffered recurrent pain with his VAS returning to nine. After two years without improvement GRS retreatment was proposed and consented to using the ZAP-X system (Figure 2); the technical goal was to cover a larger volume of the retrogasserian trigeminal nerve.

GRS technique

Pre-treatment Imaging 

After imaging with both computed tomography (CT) (Biograph Horizon PET/CT; Siemens AG, Munich, Germany) and MRI (Magnetom Symphony; Siemens AG, Munich, Germany), volumes of interest (VOIs) were contoured on top of a fused dataset within the ZAP-X treatment planning system, as previously detailed [9]. Prior to treatment, the patient is positioned in a nonrigid thermoplastic mask (Orfit High Precision Head Support system and Duon 2.4 mm U-Plast Hybrid; Wijnegem, Belgium: Orfit Industries). Thereafter, the GRS alignment process commences, involving a sequence of 3D alignment steps using non-coaxial kV x-ray images from multiple gantry angles of the patient. These images are co-registered to digitally reconstructed radiographs (DRRs) generated from the initial CT used in the treatment plan.

Treatment Planning and Delivery

During SRS, the retrogasserian portion of the trigeminal nerve was irradiated with 100 isocentric beams in a single fraction using a total of 24152.92 MU. Seventy-five gray was prescribed to the 72.20% isodose line for a single isocenter with a 5 mm collimator, and 6 mm ventral to the brainstem (Figure 1, panels D-F). A total of 99.5% of a contoured target of 0.05 cc on the trigeminal nerve was covered, with a maximum dose (isodose 100%) of 10387.81 cGy. Inverse planning was used to automatically determine beam-specific weights and defined maximum to specific VOIs while minimizing dose to other VOIs. The total treatment delivery time (plan load to completion of delivery) was 30 minutes. During GRS, the patient's position was tracked and any movement compensated at 45 seconds intervals, involving a total of 44 images.

Post-radiosurgery follow-up

**Figure 3:**
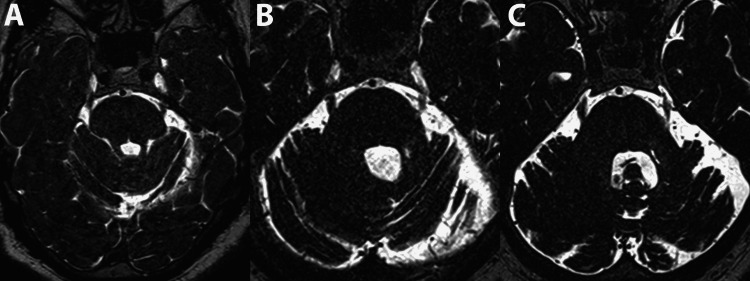
Radiographic progression. Axial FIESTA magnetic resonance imaging series from (A) initial diagnosis of trigeminal neuralgia, (B) following GKRS (Stockholm, Sweden: Elekta AB) treatment, and (C) following ZAP-X (San Carlos, CA: ZAP Surgical Systems, Inc.) GRS treatment. Atrophy within the distal left retrogasserian trigeminal nerve is noted within the post-GRS imaging. FIESTA: fast imaging employing steady-state acquisition; GKRS: Gamma Knife radiosurgery; GRS: gyroscopic radiosurgery

There were no immediate post-treatment complications and the patient was able to return home the same day. At a three-month follow-up, the patient reported a VAS of five, reduced from nine pre-GRS, and denied facial numbness. The patient maintained his anti-epileptic medication regimen but no longer required narcotics. The patient’s MRI demonstrated a region of atrophy within the retrogasserian trigeminal nerve that corresponded with the placement of the isocenter (Figure 3, panles A-C). Notably, the given patient had a history of GKRS for TN, the pre-GRS and post-GRS imaging comparison demonstrates the discernable atrophy within the distal retrogasserian trigeminal nerve.

## Discussion

This case report demonstrates the successful use of GRS for efficient and efficacious treatment of TN without any adverse effects. The patient’s VAS went from nine to five during the three-month post-treatment interval, indicating at least a partial response to treatment. While this interval is inadequate to determine long-term outcome, it serves as a benchmark for short-term success, particularly given the patient's complex medical history. The short follow-up interval and inclusion of a single patient represent significant limitations of this report. However, this first literature report on TN treatment with GRS highlights the system’s potential for treating TN with SRS.

## Conclusions

This study represents the first incidence of GRS within the literature for radiosurgical management of TN. Although requiring considerably more future investigation, this case could mark a seminal event for GRS. Such studies are clearly needed to define the long-term efficacy of this new technical approach.
